# Nitrogen and Phosphorus Retranslocation of Leaves and Stemwood in a Mature *Eucalyptus* Forest Exposed to 5 Years of Elevated CO_2_

**DOI:** 10.3389/fpls.2019.00664

**Published:** 2019-05-31

**Authors:** Kristine Y. Crous, Agnieszka Wujeska-Klause, Mingkai Jiang, Belinda E. Medlyn, David S. Ellsworth

**Affiliations:** Hawkesbury Institute for the Environment, Western Sydney University, Penrith, NSW, Australia

**Keywords:** elevated CO_2_ concentration, FACE, N:P ratio, stoichiometry, phosphorus limitation, senesced leaves, resorption

## Abstract

Elevated CO_2_ affects C cycling processes which in turn can influence the nitrogen (N) and phosphorus (P) concentrations of plant tissues. Given differences in how N and P are used by plants, we asked if their stoichiometry in leaves and wood was maintained or altered in a long-term elevated CO_2_ experiment in a mature *Eucalyptus* forest on a low P soil (EucFACE). We measured N and P concentrations in green leaves at different ages at the top of mature trees across 6 years including 5 years in elevated CO_2_. N and P concentrations in green and senesced leaves and wood were determined to evaluate both spatial and temporal variation of leaf N and P concentrations, including the N and P retranslocation in leaves and wood. Leaf P concentrations were 32% lower in old mature leaves compared to newly flushed leaves with no effect of elevated CO_2_ on leaf P. By contrast, elevated CO_2_ significantly decreased leaf N concentrations in newly flushed leaves but this effect disappeared as leaves matured. As such, newly flushed leaves had 9% lower N:P ratios in elevated CO_2_ and N:P ratios were not different in mature green leaves (CO_2_ by Age effect, *P* = 0.02). Over time, leaf N and P concentrations in the upper canopy slightly declined in both CO_2_ treatments compared to before the start of the experiment. P retranslocation in leaves was 50%, almost double that of N retranslocation (29%), indicating that this site was P-limited and that P retranslocation was an important mechanism in this ecosystem to retain P in plants. As P-limited trees tend to store relatively more N than P, we found an increased N:P ratio in sapwood in response to elevated CO_2_ (*P* < 0.01), implying N accumulation in live wood. The flexible stoichiometric ratios we observed can have important implications for how plants adjust to variable environmental conditions including climate change. Hence, variable nutrient stoichiometry should be accounted for in large-scale Earth Systems models invoking biogeochemical processes.

## Introduction

Nitrogen (N) and phosphorus (P) are arguably the two most essential macronutrients to regulate plant growth and productivity in terrestrial ecosystems (Elser et al., 2007; Harpole et al., 2011) because of their key role in many cellular functions of plant metabolism. Nitrogen has been related to plant carbon assimilation via the many photosynthetic proteins (Field and Mooney, 1986; Evans, 1989; Reich et al., 2008), whilst P is a central component of energy metabolism (ATP and P_i_) and takes part in the formation of the key macromolecules such as nucleic acids, phospholipids, ribosomes and sugar phosphates (Bieleski, 1973; Jacob and Lawlor, 1991; Rao and Terry, 1995). However, both of these essential nutrients are often in short supply relative to a plant’s growth requirements, especially in forest ecosystems on nutrient-poor soils (Vitousek and Farrington, 1997; Vitousek et al., 2010). Scarcity of N and P can affect the metabolic performance of plants (Sterner and Elser, 2002; Reich and Oleksyn, 2004; Ågren et al., 2012). Most native ecosystems exhibit strong N- or P-limitation, or a co-limitation of both as N and P tend to co-vary in nature (Sterner and Elser, 2002). However, N-limitation can occur more frequently than P-limitation in geologically young soils (LeBauer and Treseder, 2008); whereas P-limitation tends to be more prevalent in old, highly weathered soils (Walker and Syers, 1976; Vitousek et al., 2004; Turner et al., 2013). In green leaves, N and P concentrations are important not only because of their functional relationships to photosynthetic assimilation rates and growth (Aerts and Chapin, 2000; Wright et al., 2004; Ågren, 2008), but also because they may regulate carbon (C) and nutrient cycles and food webs in terrestrial ecosystems (Chapin et al., 2011).

Approximately one-third of the world’s soils are estimated to be P-limited based on the geographical extent of soil types (Yang and Post, 2011), and the high N:P ratios in leaves of plants growing on such soils (Reich and Oleksyn, 2004; Cleveland et al., 2011; Goll et al., 2012). The Australian continent is well-known for its geologically old landscapes with low P availability in soils (Beadle, 1966; Lambers et al., 2010; Kooyman et al., 2017), related to the parental materials that lack renewal from glaciation and tectonic action (Hopper, 2009; Augusto et al., 2017). Hence, many but not all landscapes on this continent may be particularly low in P (Kooyman et al., 2017).

Retranslocation of nutrients from leaves during senescence is a strategy to efficiently retain P (Escudero et al., 1992; Aerts, 1996; Killingbeck, 1996). This retranslocation by resorption serves to withdraw nutrients from leaves prior to abscission for later redeployment in developing tissues. While resorption can occur throughout the lifespan of the leaf (Ackerly and Bazzaz, 1995; Wright and Westoby, 2003), it is especially an intrinsic process of leaf senescence that occurs before leaf abscission. The nutrient resorption efficiency or retranslocation rate is the ratio determined by comparing the nutrient concentration in senesced leaves with the nutrient concentrations in fully expanded, mature green leaves (Killingbeck, 1996). The extent to which N and P are retranslocated and re-used depends on the plant nutrient status (Aerts and Chapin, 2000). Generally, with decreasing nutrient availability in an ecosystem, the amount of resorption of both N and P tend to increase (Reed et al., 2012; Vergutz et al., 2012) and this ratio could be an indicator of which nutrient is most limiting in a given ecosystem (de Campos et al., 2013).

Given that N and P availability control the global carbon-cycle response to environmental changes (van Groenigen et al., 2006; Matear et al., 2010; Wassen et al., 2013), it is important to investigate both elements and their stoichiometric balance. N and P concentrations in green leaves are typically correlated both within and across plant species and ecosystems (McGroddy et al., 2004; Hessen et al., 2007), leading to a narrow stoichiometric range for N:P (Elser et al., 2007). As low P availability in the soil tends to decrease leaf P concentrations (Cleveland et al., 2011; Hidaka and Kitayama, 2011; Marklein and Houlton, 2012), foliar N:P ratios increase. Moreover, N-use efficiency tends to decrease as plants had higher N:P ratios (Crous et al., 2017). However, plant species differ in their ability to maintain a constant N:P ratio under variable supply of both nutrients (Sterner and Elser, 2002; Güsewell, 2004) and it is unclear how flexible this stoichiometry can be, especially in response to climate change factors such as elevated atmospheric CO_2_. Several studies have reported a decrease in leaf N:P ratios in response to elevated CO_2_ (Dijkstra et al., 2012; Liu et al., 2013; Deng et al., 2015; Yue et al., 2017) although some species did not show such a change (Milla et al., 2006; Liu et al., 2013). The decrease in N:P in response to elevated CO_2_ has been explained by the observation that N concentrations may be more sensitive to elevated CO_2_ than P-concentrations, something that has primarily been observed in N-limiting ecosystems (Feng et al., 2015; Huang et al., 2015). However, the importance of P dynamics in elevated CO_2_ is less clear, and the assumption of a homeostatic N:P ratio in elevated CO_2_ due to similar proportional N and P responses needs to be tested (Ågren, 2008; Goll et al., 2012; Zhang et al., 2014). Given the potential for elevated CO_2_ to change stoichiometric ratios (Sardans et al., 2012; Deng et al., 2015; Yuan and Chen, 2015), it is critical to understand how elevated CO_2_ affects plant N:P ratios to evaluate nutrient limitations in terrestrial ecosystems in the future.

In this study, we hypothesized that (1) P-retranslocation would be higher than N-retranslocation in a mature woodland on the Sydney Cumberland Plain, given that there is evidence it is a P-limited ecosystem (Crous et al., 2015; Ellsworth et al., 2017) and (2) that elevated CO_2_ would reduce N:P ratios in leaves and/or wood due to the greater mobility of N in leaves and the large body of evidence showing strong N reductions under elevated CO_2_ (Feng et al., 2015). To test these, we determined nutrient concentrations and their retranslocation of *Eucalyptus tereticornis* leaves and wood from a mature woodland exposed to long-term elevated CO_2_ at *Eucalyptus* Free-Air CO_2_ Enrichment experiment (EucFACE). We investigated both spatial and temporal variations in leaf P and N concentrations in this ecosystem and tested whether long-term elevated CO_2_ affected P concentrations and N:P ratios in green leaves, senesced leaves and wood.

## Materials and Methods

### Site Description for EucFACE

The *Eucalyptus* Free-Air CO_2_ Enrichment (EucFACE) experiment is located in a remnant patch of native Cumberland Plain woodland on an ancient alluvial floodplain in western Sydney (Australia, 33° 37′S, 150° 44′E, 30 m a.s.l.) and has been unmanaged for over 70 years. The soil is a loamy sand (Podosol in the Australian Soil Classification system) with >75% sand content changing into sandy clay loam with >30% silt and clay between 0.5 – 2 m depth, transitioning to sandy clay beyond 2 m depth down to the water table at 13 m. The surface soil pH is slightly acidic (pH_water_ ∼5.2 ± 0.1, lower than reported in Crous et al., 2015). Total P concentration in the soil done by Aqua Regia assay was 76 mg kg^-1^ (0–10 cm soil depth, Drake et al., 2016), with available P much lower than this at <10 mg kg^-1^ (Bray extraction method used in Hasegawa et al., 2016). The soil is nutrient poor, and a strong limitation of P on tree growth at the site has been demonstrated (Crous et al., 2015). The open woodland (600–1000 trees ha^-1^) has a mean annual temperature of 17°C and mean annual precipitation of ∼800 mm (1881 – 2018, Bureau of Meteorology, station 067105 in Richmond, NSW Australia^1^). The vegetation is dominated by *Eucalyptus tereticornis* Sm. in the overstory with a few scattered individuals of *E. amplifolia* Naudin, while the understory is dominated by *Microlaena stipoides* (Labill.) R.Br. (a C_3_ grass) and some shrubs (see Pathare et al., 2017).

The Free-Air CO_2_ enrichment site consists of six 25 m diameter circular plots (hereafter referred to as ‘rings’) from which 32 vertical pipes are extended above the forest canopy, as described previously in Gimeno et al. (2016) and Duursma et al. (2016). Three rings were exposed to an elevated [CO_2_] treatment (ambient +150 μmol mol^-1^) while the other three rings were used as control plots representing the ambient [CO_2_] treatment (∼400 μmol mol^-1^), with identical infrastructure and instrumentation as the treatment plots. Pre-diluted CO_2_ is released into the treatment rings in a computer controlled system aiming for a CO_2_ mole fraction of 150 μmol mol^-1^ above ambient since February 2013.

### Leaf and Wood Sampling

Green fully expanded mature leaves were collected from three (co-)dominant trees in each plot using multiple 36 m tall canopy-access cranes (Jaso crane J-4010, Jaso S.L., Idiazabal, Spain). Leaves were collected from the top of the tree crowns at 17–23 m height, depending on the height of dominant trees within the plots. Measurements of leaf N and leaf P on fully expanded mature green leaves started in May 2012 before CO_2_ fumigation at EucFACE was turned on. Additional leaves were sampled several times per year from 2013–2018, viz. Jan./Feb., April/May, and Sept./Oct., totaling 473 green leaf samples. This roughly corresponded to 1 or 2 subsample leaves per tree and three trees × 6 plots ×3–4 times of year ×5 ½ years. In a randomized design for the overall experiment, degrees of freedom were allocated for true replicates (plots) and subsamples within plots were averaged. Leaf life span in *E. tereticornis* was on average 13 months (Duursma et al., 2016) with newly flushed leaves here defined as less than 90 days old and mature leaves defined as more than 90 days old, consistent with Wujeska-Klause et al. (2019) and based on phenological observations of timing of leaf flushing (Ellsworth, unpublished). For most of the year, leaves were mature and older than 90 days, but during summer when canopy turnover occurred, two leaf age classes were collected (when available) including newly flushed fully expanded leaves and old mature leaves. Upon collection, leaves were placed on ice and measured for leaf area, after which they were freeze-dried for at least 72 h to determine leaf mass per area (LMA, g m^-2^), and then prepared for chemical analyses when ground to a fine powder. All green leaves used to compute retranslocation were live, mature green leaves collected from the upper canopy toward the end of their lifespan (∼360–410 days old).

Leaf litter was collected monthly in ∼0.2 m^2^ circular fine-mesh traps at eight random locations per ring (Duursma et al., 2016). Litter was sorted into leaf, twigs, bark, and other material, then oven-dried at 40°C and weighed. Given that canopy turnover occurred in summer, a subsample of senesced leaves was analyzed from 2–3 traps per ring for total N and total P concentrations for the summer months of 2013–2018, totaling 105 samples. Some senesced leaves were also collected from individual trees during canopy turnover in the summers of January 2014 and February 2015, in which case the values were averaged per ring with the litter trap nutrient values. Senesced leaf nutrient concentrations were also used to compute retranslocation, calculated as the fraction of nutrients removed from its senescing leaves compared with how much is in its adult leaves before leaf abscission:

$$\mathrm{Retranslocation}=\frac{\left([green\;leaf]-[senesced\;leaf]\right)}{\left[green\;leaf\right]}$$

Wood was sampled in November 2015 at the base of the stem (about breast height) in three (co-) dominant trees per ring. Sapwood was defined as the outer 20 mm of wood beneath the bark, and was sampled for a 4 cm × 2 cm patch using a chisel. Gimeno et al. (2018) determined that the outer 20 mm comprised most of the sapwood in dominant and co-dominant trees in the stand. Heartwood was sampled at one timepoint outside one ring in the middle of the EucFACE site. Heartwood was judged on the basis of wood color and density which distinguishes it from sapwood in this species. Heartwood could not be collected from within the experimental rings due to the long-term nature of the experiment.

### Elemental Analyses of Leaf Nitrogen and Phosphorus

Mass-based leaf N (N_m_) and C concentrations were analyzed with the Dumas combustion technique using elemental analyzers (TruSpec micro, LECO Corp., St. Joseph, MI, United States; and FLASH EA 1122 Series CHN analyzer, Thermo-Finnigan, Waltham, MA, United States). Total P concentrations of leaves were determined via a standard Kjeldahl digestion procedure using 3 mL of sulphuric acid (pure H_2_SO_4_) and 2 mL of hydrogen peroxide (H_2_O_2_, 30%). The amounts of dried material digested varied depending on how much total P was expected in the sample. For example, we used 55 mg for green leaves, 250 mg for senesced leaves and 450 mg for sapwood. The total P concentrations of the Kjeldahl digests were colorimetrically analyzed at 880 nm after a molybdate reaction in a discrete analyzer (AQ2, SEAL Analytical, Ltd., Milwaukee, WI, United States and EPA135 method). Elemental analyses of other macronutrients are reported in Crous et al. (2015). Sapwood was analyzed by inductively coupled plasma optical emission spectroscopy (ICP-OES, Perkin-Elmer, Waltham, MA, United States) for total P and via combustion for total N (FLASH EA 1122 Series CHN analyzer, Thermo-Finnigan, Waltham, MA, United States) for 2–3 trees per ring. Heartwood was also analyzed for total P concentration with the same method and used to calculate P retranslocation in wood across CO_2_ treatments. For all chemical analyses we ran internal standards, using NIST Standard Reference Material 1515 (United States National Institute of Standards and Technology, Bethesda, MD, United States) for quality control purposes. Area-based N (N_a_; g m^-2^) and P (P_a_; g m^-2^) content were calculated based on P_m_ and N_m_ concentrations and multiplied by the leaf per mass area (LMA; g m^-2^).

### Statistical Analyses

All statistical analyses were performed in R (R Core Team, 2018, v. 3.5.1). Variables were tested for normality to conform with linearity assumptions before further statistical analyses. Transformations were applied, where necessary to achieve a normal distribution: P_m_ and C:N were both log-transformed in green leaves. Over this 6-year dataset, we only used campaigns, where both N and P concentrations had been measured, and as such this dataset is slightly different from the dataset used in Wujeska-Klause et al. (2019). Most findings regarding N are consistent with this previous paper, and our analyses here additionally focus on P, including evaluating annual variation.

We conducted a set of mixed model analyses on green leaves with CO_2_, Year and Age as fixed factors and tree nested within ring as a random factor using the ‘lmer’ function from lme4 package (Bates et al., 2015). We also analyzed leaf P concentrations using Year as a continuous variable and obtained similar results. Differences between means were tested using Tukey HSD *post hoc* test [‘glht’ from multcomp package, Hothorn et al. (2008)]. All analyses used tree-specific data for each measurement campaign. Means and standard error bars of CO_2_ treatment effects were computed based on ring averages to reflect the correct number of replicates (*n* = 3). Mixed model analyses of senesced leaves included CO_2_ and Year as fixed factors with Ring as a random factor, sampled each year in February. February was chosen because most leaf litter falls in the summer months as the canopy is turning over. Given the destructive nature, we sampled sapwood from trees at the site at a single time. Thus, we performed 2-sample *t*-tests to evaluate the CO_2_ effect on sapwood C, N and P concentrations along with their ratios. Data are stored on Western Sydney’s data archive (https://hiev.uws.edu.au/) and are publicly available at https://doi.org/10.6084/m9.figshare.7898048.v1.

## Results

### Effects of Leaf Age and Its Interaction With Elevated CO_2_

**FIGURE 1:**
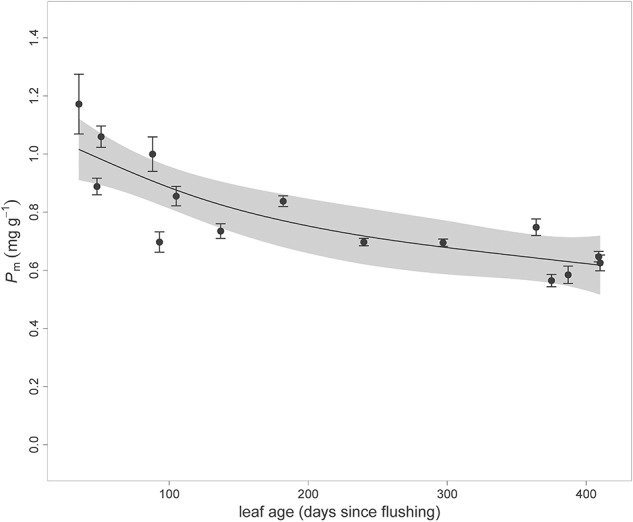
Means and standard errors of mass-based leaf phosphorus concentrations (P_m_) since leaf flushing for each campaign across years, showing a decline in P concentrations over the lifetime of leaves (expressed as days since flushing where leaves are ‘new’ when <90 days). The line shows the trend fit using a generalized additive model (GAM), and the gray area shows the 95% confidence interval around the mean decline in P_m_. Mass-based N as a function of leaf age can be found in Wujeska-Klause et al. (2019).

There was no elevated CO_2_ effect on mass-based leaf P (P_m_) concentrations. There was a strong decline in green leaf P_m_ as leaves aged (*P* < 0.0001, Tables 1, 2), with P_m_ in old green leaves being 32% lower compared to newly flushed leaves across years (<90 days old) (Table 2). Over the total leaf lifespan, there was a fairly steady decline in mean P_m_ concentrations (i.e., on a continuous leaf age basis expressed as days since flushing across years) from recently flushed leaves until leaves over 400 days old, shortly before abscission (Figure 1). Across years, related to the Age effect on leaf P, both N:P and C:P ratios also exhibited significant leaf age effects. N:P ratios increased by 42% in older leaves compared to newly flushed leaves (*P* < 0.0001), where C:P ratio was 45% higher in older leaves than new leaves (*P* < 0.0001, Table 2).

**Table 1:** Mixed model Anova results with F-statistic of green upper canopy leaves over 5 years with CO_2_, Age and Year as fixed factors and tree nested within ring as random factors.

		F-statistic and significance				
		
Source	df	P_m_ (log)	N_m_	N:P	C:P	C:N (log)
CO_2_	1	0.03	1.41	0.57	0.0007	0.81
Age	1	**289.15^∗∗∗^**	0.95	**275.34^∗∗∗^**	**280.13^∗∗∗^**	2.37
Year	5	**8.63^∗∗∗^**	**15.73^∗∗∗^**	**19.70^∗∗∗^**	**17.70^∗∗∗^**	**17.23^∗∗∗^**
CO_2_ x Age	1	0.54	**13.65^∗∗∗^**	**5.36^∗^**	0.44	**12.81^∗∗∗^**
CO_2_ x Year	5	0.77	0.63	0.67	1.02	0.82
Age x Year	3	**13.33^∗∗∗^**	**8.41^∗∗∗^**	**9.49^∗∗∗^**	**10.90^∗∗∗^**	**10.37^∗∗∗^**
CO_2_ x Age x Year	3	0.64	0.64	0.12	0.53	0.51


*df represents the degrees of freedom, where two years did not have the newly flushed age class resulting in a reduction of df from 5 to 3 for the interactions. The variables are as follows: mass-based phosphorus in mg g^-*1*^ (P_*m*_), mass-based nitrogen in mg g^-*1*^ (N_*m*_), the ratio of N concentration to P concentration (N:P), the ratio of C concentration to P concentration (C:P), the ratio of C concentration to N concentration (C:N). P_*m*_ and C:N were log-transformed for this analysis. Significant F-statistics are highlighted in bold with significance level indicated as ^∗∗∗^ for *P* < 0.0001, ^∗∗^ for *P* < 0.01, ^∗^ for *P* < 0.05.*

**Table 2:** Means ± std. errors across years for mass-based phosphorus (P_m_), mass-based nitrogen (N_m_), the ratio between N and P (N:P), the ratio between C and P (C:P), the ratio between C and N (C:N) and the retranslocation fraction for P (P retrans) and N (N retrans) in ambient and elevated CO_2_ in newly flushed and mature green leaves (>90 days since flushing), senesced leaves and sapwood.

	Means across years ( ± std. error)								
	
	P_m_ (mg g^-1^)	N_m_ (mg g^-1^)	N:P (g N g^-1^ P)	C:P (g C g^-1^ P)	C:N (g C g^-1^ N)	P retrans (dimensionless)		N retrans (dimensionless)	
**New green leaf**									
Ambient CO_2_	1.04 ± 0.05	17.0 ± 0.8	17.2 ± 1.1	510 ± 19	31.0 ± 1.5				
Elevated CO_2_	1.02 ± 0.06	15.1 ± 0.6	15.4 ± 1.0	521 ± 25	34.7 ± 1.5				
**Mature green leaf**									
Ambient CO_2_	0.70 ± 0.02	16.0 ± 0.3	23.1 ± 0.4	741 ± 21.4	31.9 ± 0.68	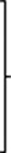	Ambient CO_2_	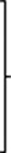	Ambient CO_2_
Elevated CO_2_	0.70 ± 0.02	15.8 ± 0.3	23.1 ± 0.5	737 ± 18.6	32.0 ± 0.49		0.52 ± 0.03		0.29 ± 0.04
**Senesced leaf**									
Ambient CO_2_	0.31 ± 0.01	11.0 ± 0.6	35.4 ± 1.4	1740 ± 107	49.6 ± 2.6		Elevated CO_2_		Elevated CO_2_
Elevated CO_2_	0.34 ± 0.02	11.3 ± 0.6	34.6 ± 1.1	1692 ± 107	49.0 ± 2.8		0.49 ± 0.04		0.28 ± 0.05
**Sapwood**									
Ambient CO_2_	0.14 ± 0.02	4.8 ± 0.9	35.6 ± 2.1	3705 ± 702	101.6 ± 14.7	Across CO_2_		-	
Elevated CO_2_	0.12 ± 0.01	5.8 ± 0.7	48.4 ± 1.1	3837 ± 469	81.3 ± 9.6	0.82			


*To calculate foliar retranslocation rates, both mature green leaves and senesced leaves were averaged per ring across six years whereas wood was measured during one timepoint.*

Leaf N_m_ did not differ between CO_2_ treatments or across leaf age classes, but there was a significant CO_2_ by Age effect in which newly flushed leaves reduced leaf N by 11% in elevated CO_2_; whereas old leaves had similar leaf N concentrations in both CO_2_ treatments, consistent with Wujeska-Klause et al. (2019). Related to this CO_2_ by Age interaction in leaf N, both N:P and C:N ratios also exhibited CO_2_ by Age interactions. The ratio N:P declined by 9% (*P* = 0.02), while C:N increased by 12% (*P* = 0.0004) in newly flushed leaves in elevated CO_2_ whereas older leaves did not show a CO_2_ treatment difference for either variable (Tables 1, 2).

Area-based P (P_a_) and N (N_a_) did not differ between CO_2_ treatments, although N_a_ showed a marginal CO_2_ by Age effect (*P* = 0.07, Supplementary Table S1) where new leaves were 5% lower in elevated CO_2_ compared to ambient CO_2_. This pattern is in the same direction as N_m_ concentrations and suggests that there was no dilution effect due to elevated CO_2_, as reported in Wujeska-Klause et al. (2019). Both P_a_ and N_a_ exhibited significant differences between leaf age classes where N_a_ was 23% lower in new leaves (*P* < 0.0001) due to a 23% lower LMA when compared with old leaves (*P* < 0.0001), while P_a_ was about 12% higher in new compared to old leaves (*P* = 0.0001). Leaf mass per area ratio (LMA) was not significantly different between CO_2_ treatments but was trending toward a 5% higher LMA in elevated CO_2_ (*P* = 0.08, Supplementary Table S1).

### Year-Effects on P and N in Green Leaves

**FIGURE 2:**
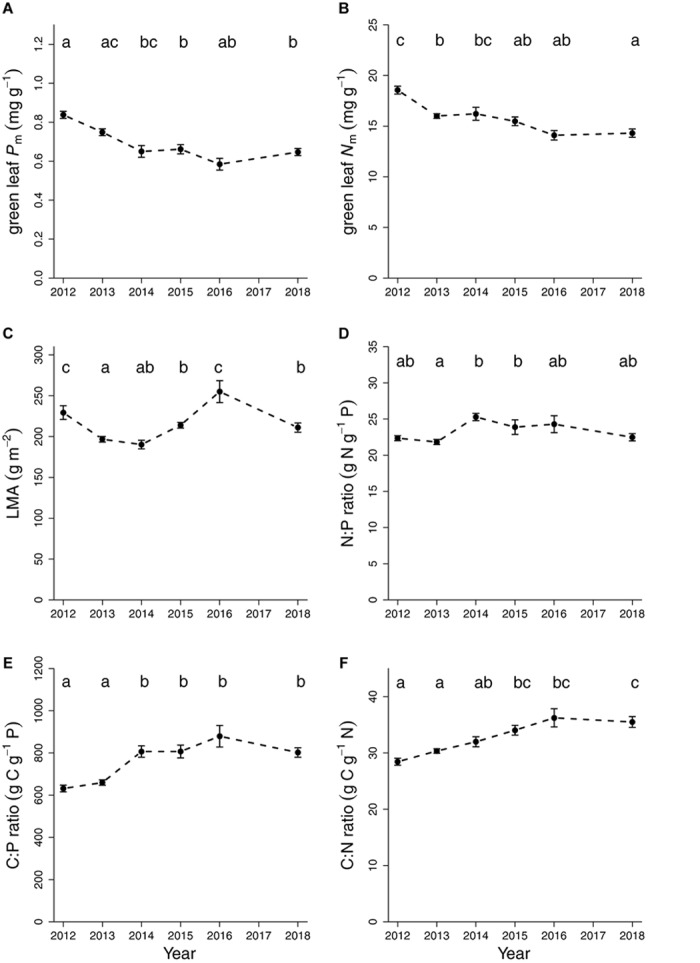
Annual variation in chemistry and structure for mature green leaves averaged across measurement campaigns for **(A)** mass-based phosphorus in mg g^-1^, **(B)** mass-based nitrogen in mg g^-1^, **(C)** LMA in g m^-2^, **(D)** N:P ratio, **(E)** C:P ratio and **(F)** C:N ratio. Data are means and standard errors. There were no mature green leaves in February 2017 due to early flushing.

All variables showed highly significant differences among years (*P* < 0.0001) and significant interactions of Year ×Age (Table 1). These Year by Age interactions generally showed that newly flushed leaves exhibited more variable responses across years, likely due to the time of measurement after they flushed, which differed between 35 and 88 days. By contrast, older mature leaves were more steady in their response over the years, regardless of sampling time. Therefore, we focused on mature leaves only to evaluate the year effects (Figure 2).

*Post hoc* Tukey tests indicated that P_m_ concentrations in mature leaves in the first 2 years (2012 and 2013) were higher compared to the latter years. As there was no effect of elevated CO_2_, this decline was similar in both CO_2_ treatments. Hence, across CO_2_ treatments P_m_ concentrations in green mature leaves declined from 0.79 ± 0.03 mg g^-1^ (2012 and 2013 together) to 0.64 ± 0.02 mg g^-1^ in later years (2014, 2015, 2016, 2018) (Figure 2A). This pattern across years was similar regardless of sampling time (Supplementary Figure S1).

Leaf N_m_ concentrations also declined across years but the trend was less clear compared to leaf P_m_. While *post hoc* tests indicated significant differences between 2012, 2013 and 2018, there were no differences between 2013 and 2016 for N_m_ in mature green leaves. Across CO_2_ treatments, leaf N_m_ declined from 18.5 ± 0.4 mg g^-1^ in 2012 to 15.4 ± 0.4 mg g^-1^ in 2013–2016, and then to 14.3 ± 0.4 mg g^-1^ in 2018 (Figure 2B).

Given the differences between years in N and P concentrations of mature green leaves, the N:P, C:P and C:N ratios were also different across years (*P* < 0.0001) and no CO_2_ effects were observed (*P* > 0.1, Table 1). While N:P was highest in 2014 and 2015 (24.6 ± 0.8) and different from the N:P in 2013 (21.8 ± 0.4), differences in other years were subtle and non-significant. Hence, the overall N:P ratio across years was 23.1 ± 0.5 in mature leaves (Figure 2C and Table 2). The C:P ratio was higher in later years compared to earlier years (2012, 2013), being 824 ± 32 versus 645 ± 14, respectively, likely reflecting the reduced leaf P concentrations in the later years (Figure 2D). Across CO_2_ treatments, C:N increased over the years reflecting declining leaf N concentrations with very clear trends contrasting earlier (2012–2013: 29.4 ± 0.5) to later years (2015–2018: 35.3 ± 1.1).

On an area-basis, N_a_ and P_a_ declined in similar fashion as their mass-based variables. Both P_a_ and N_a_ had higher concentrations in 2012 compared to all other years. P_a_ also showed a CO_2_ by year interaction (*P* = 0.05, Supplementary Table S1) because in some years P_a_ was higher in elevated CO_2_ while in other years it was lower compared to ambient CO_2_ within a given year. These small variations did not result in any overall CO_2_ effect on P_a_. LMA varied over the years averaging at 206 ± 3 g m^-2^ in old leaves with the lowest value in 2014 (175 ± 6 g m^-2^) and the highest in 2016 (240 ± 3 g m^-2^) (Figure 2C). These values are consistent with Wujeska-Klause et al. (2019).

### P and N Concentrations in Senesced Leaves and Their Retranslocation Rate

**FIGURE 3:**
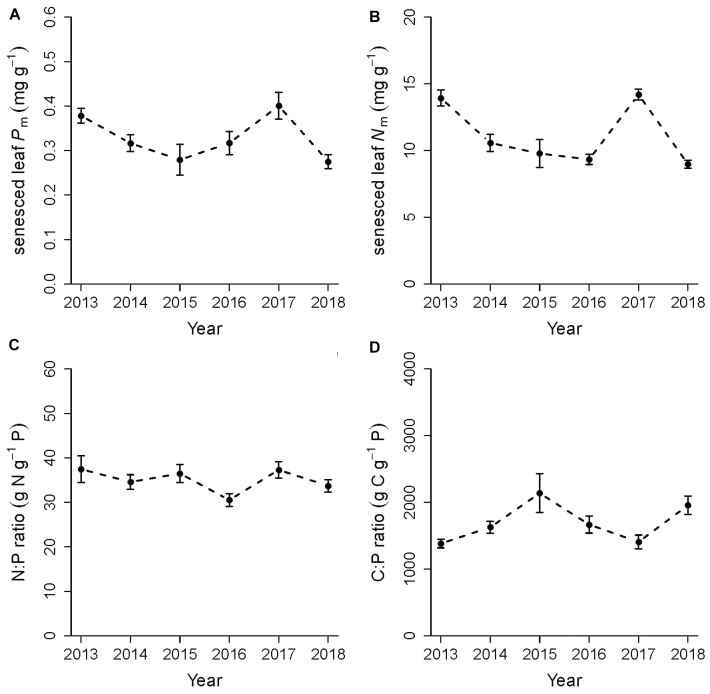
Annual variation in chemical composition of senesced leaves collected from litter traps in February of each year (2013–2018) for **(A)** mass-based phosphorus in mg g^-1^, **(B)** mass-based nitrogen in mg g^-1^, **(C)** N:P ratio and **(D)** C:P ratio. Data are means and standard errors.

Across years and CO_2_ treatments, the concentrations of P and N in senesced leaves were, respectively, 0.32 ± 0.04 mg g^-1^ and 11.1 ± 1.5 mg g^-1^ on average. Mixed model analyses indicated no effects of elevated CO_2_ on P or N concentrations in senesced leaves, but as with green leaves, there were significant year effects in which leaf P and N concentrations varied between years (Figure 3 and Table 3). Both N and P concentrations in senesced leaves were lower in 2014, 2015, 2016 and 2018 compared to 2013 and 2017 (*P* < 0.05, Tukey test). Senesced leaf C was on average 51%. All ratios also varied across years but without a clear directional trend. Although senesced leaf N:P ratio has a weak year effect (*P* = 0.046), *post hoc* comparison did not indicate significant differences. Across years, the N:P ratio was 35.0 ± 2.9 (Figure 3 and Table 2). Similarly for C:P in senesced leaves, there were no significant *post hoc* comparisons with the C:P ratio being 1716 ± 259 across years. C:N mirrored the pattern in senesced leaf N with 2013 and 2017 being similar whereas the ratio increased in the middle years.

**Table 3:** Mixed model Anova table with F-statistic of senesced leaves over five years with CO_2_ and Year as fixed factors and ring as random factor.

	F-statistic and significance							
		
Source	df	P_m_	N_m_	N:P	C:P	C:N	P retrans	N retrans
CO_2_	1	2.79	1.13	0.50	1.15	0.92	0.25	0.045
Year	5	**4.59^∗∗∗^**	**16.57^∗∗∗^**	**2.36^∗^**	**4.66^∗∗∗^**	**12.82^∗∗∗^**	1.56	**6.86^∗∗^**
CO_2_ x Year	5	0.95	0.20	0.56	0.27	0.10	1.45	0.98


*df represents the degrees of freedom and the variables are as follows: mass-based phosphorus in mg g^-*1*^ (P_*m*_), mass-based nitrogen in mg g^-*1*^ (N_*m*_), the ratio of N to P concentration (N:P), the ratio of C to P concentration (C:P), the ratio of C to N concentration (C:N) and retranslocation of phosphorus (P retrans) and nitrogen (N retrans). C:N was sqrt-transformed to satisfy normality assumptions. Significant F-statistics are highlighted in bold with significance level indicated as ^∗∗∗^ for *P* < 0.0001, ^∗∗^ for *P* < 0.01, ^∗^ for *P* < 0.05.*

**FIGURE 4:**
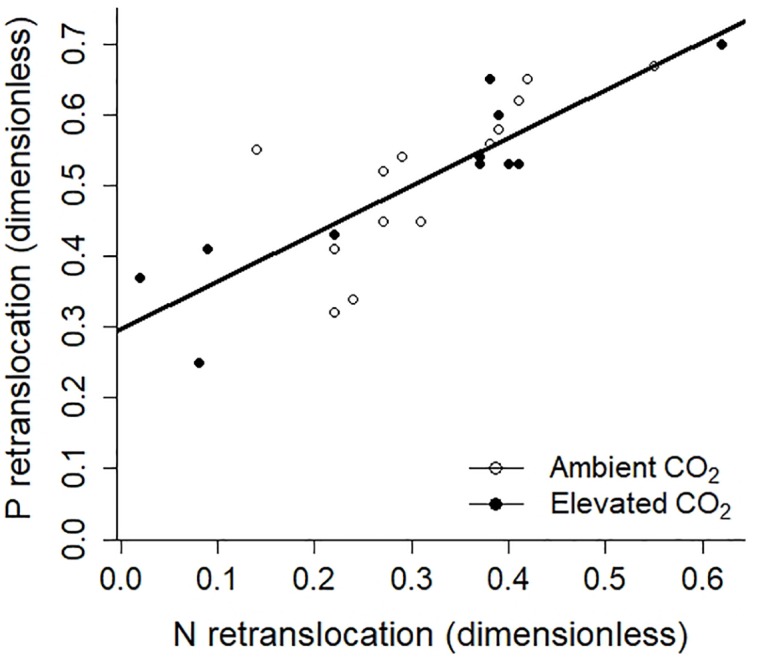
Linear relationship between P retranslocation and N retranslocation across 5 years in ambient (open symbols) and elevated (closed symbols) CO_2_ treatments, expressed as a fraction (dimensionless). The relationship was described as *Y* = 0.68 ^∗^ N retranslocation fraction + 0.30, *R*^2^ = 0.66 and *P* < 0.0001.

The retranslocation rate or resorption efficiency was calculated using N and P concentrations in the oldest green leaves and comparing these with the respective concentrations in senesced leaves (leaf age > 360 days, last 4 ticks in Figure 1). Both green leaves and senesced leaves were collected in the January-February period of each year (except for January 2017 where no old leaves were available). On average, leaves re-translocated 50.1 ± 2.4% P and 28.7 ± 3.0% N across years (means ± s.e. across years; Table 2). While there was no year effect on P retranslocation, N retranslocation was different across years because N retranslocation in 2013 was much lower compared to the other years. Overall, there was a strong positive relationship (*R*^2^ = 0.66) between N and P retranslocation across the years at the site with no differences regarding CO_2_ treatment (Figure 4).

### Leaf P, N and C Concentrations in Wood

**FIGURE 5:**
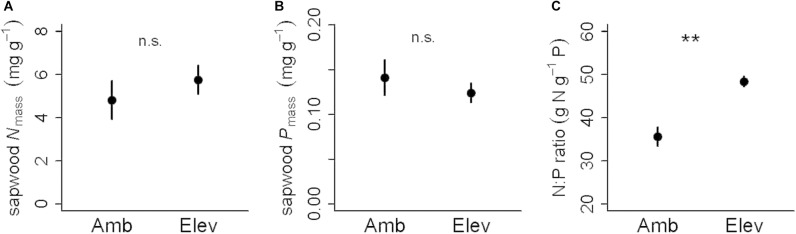
Means and standard errors for chemical composition of sapwood in ambient (Amb) and elevated (Elev) CO_2_ treatments for **(A)** mass-based nitrogen concentration in mg g^-1^, **(B)** mass-based P concentration in mg g^-1^ and **(C)** N:P ratio. While N and P concentrations did not differ between CO_2_ treatments (n.s. in panels **A** and **B**), the N:P ratio in wood was significantly increased in elevated CO_2_ compared to ambient CO_2_ at *P* < 0.01 (^∗∗^ in panel **C**).

Concentrations of P and N in sapwood were 50–60% lower than concentrations in senesced leaves. Neither wood N nor P concentrations were statistically different between CO_2_ treatments, with averages of 5.3 ± 0.8 mg g^-1^ (N) and 0.13 ± 0.01 mg g^-1^ (P) (Table 2). In sapwood, the C concentration was 45%. The total P concentration in heartwood was 0.023 ± 0.005 mg g^-1^, so assuming heartwood was representative across treatments this resulted in a 82% P retranslocation rate from sapwood (Table 2). Whereas C:N and C:P in sapwood were not significantly different between CO_2_ treatments (*P* > 0.3), there was a significant increase in sapwood N:P ratios in elevated CO_2_ compared to ambient CO_2_ (*P* = 0.006). Here, sapwood N:P ratios increased to 48 ± 1 in elevated CO_2_ compared to 36 ± 2 in ambient CO_2_ (Figure 5 and Table 2). Although both wood N and P concentrations were not significantly different between CO_2_ treatments (*P* > 0.2), the CO_2_ effect on N:P appears to be underpinned by a 20% increase in mean N concentrations in sapwood in elevated CO_2_, and a smaller decrease in wood P concentration in elevated CO_2_ (Figure 5). These together led to the observed CO_2_ effect on wood N:P ratio.

## Discussion

In this mature *Eucalyptus* forest, long-term elevated CO_2_ exposure did not significantly change N or P concentrations in mature green leaves (*P* > 0.10, Table 1), though such changes have been observed by others (Sardans et al., 2012) and were initially hypothesized. In contrast to our study, several studies reported decreased N:P ratio in elevated CO_2_, in part as a consequence of observed lower leaf N in elevated CO_2_ relative to leaf P (Sardans et al., 2012; Liu et al., 2013; Deng et al., 2015; Huang et al., 2015). However, severely nutrient-limited bog sites also did not show a CO_2_ effect on foliar N and P concentrations (Milla et al., 2006). When N is limiting in an ecosystem, plants can reduce leaf N in elevated CO_2_ as has been frequently observed (Ainsworth and Rogers, 2007; Liu et al., 2013; Deng et al., 2015). We observed a transient elevated CO_2_ effect on leaf N in newly flushed leaves but not in older, mature leaves. This is shown by the significant interaction between elevated CO_2_ and Age for N_m_, N_a_, N:P and C:N ratios in our study (Table 1), but not for P_m_. Hence, elevated CO_2_ reduced N:P in newly flushed leaves but N:P was similar in both CO_2_ treatments in mature green leaves because leaf N concentrations declined more strongly with age x CO_2_ (Wujeska-Klause et al., 2019) compared to leaf P concentrations across the lifetime of leaves in this evergreen eucalypt forest (Figure 1). Wujeska-Klause et al. (2019) reported non-significant differences in carbohydrates between CO_2_ treatments and no strong evidence of structural dilution in elevated CO_2_. Therefore, these processes unlikely influenced N_m_ and P_m_. Hence, any elevated CO_2_ effects detected for leaf N in newly flushed leaves diminished as leaves matured beyond three months since flushing, noting that the *Eucalyptus* leaf longevity at our site was about a year (Duursma et al., 2016).

The EucFACE mature woodland is P-limited, indicated by high N:P ratio both for green and senesced leaves (Table 2). These N:P ratios suggest a clear P-limitation at the site (Koerselman and Meuleman, 1996), consistent with interpretations from Crous et al. (2015). Moreover, P- retranslocation was about 50%, almost twice as high as N-retranslocation (28%), so greater retranslocation of a more scarce nutrient is further evidence of P-limitation (Wright and Westoby, 2003). There was proportionally more P in green leaves being removed prior to senescence than N, shown by the higher N:P ratio (N:P of 35) in senesced leaves compared to green leaves. The higher resorption efficiency of P than N represents a key process that can generate differences between foliar N and P concentrations as well as different N:P ratios in leaves and wood (Sardans and Peñuelas, 2013).

The resorption proficiency, defined as the level of nutrient concentrations in senesced leaves (Wright and Westoby, 2003), was 0.32 ± 0.04 mg g^-1^ in our study. Killingbeck (1996) reported a biochemically complete resorption of P when the P concentration of senesced leaves in evergreen species is less than 0.4 mg g^-1^. Therefore, P resorption in *E. tereticornis* leaves can be considered ‘complete,’ suggesting that leaves did not only resorb all soluble P but also hydrolyzed P in lipids and nucleic acids before senescence (Hidaka and Kitayama, 2011). Hydrolyzing enzymes as phospholipase and ribonucleases have a certain cost in terms of N requirements in synthesis (Fischer, 2007). However, when P availability is low, the balance between N cost for these enzymes and the benefit of P gain by resorption (Hidaka and Kitayama, 2011) is apparently in favor of P resorption in this ecosystem.

When a particular nutrient is limiting plant growth, there are several mechanisms to mitigate this nutrient limitation, two of which are increased uptake or increased nutrient-use efficiency. In N-limited pine plantation in Duke Forest, increased N demand from faster growth in elevated CO_2_ was largely accommodated via increased N uptake from soils (Phillips et al., 2012), while N retranslocation only played a minor role in this pine ecosystem (Finzi et al., 2002). In contrast, a small increase in P mineralization was observed in the elevated CO_2_ soil after the first 3 months of CO_2_ exposure (in 2012) in this *Eucalyptus* forest ecosystem after which no significant CO_2_ treatment effects were found on P mineralization rates (Hasegawa et al., 2016). There is no evidence that this transient effect influenced canopy P concentrations (Figure 2), arguing against increased P uptake in the trees in elevated CO_2_. A more definitive analysis would require assembling a full P budget for this ecosystem. However, high retention of foliar P across CO_2_ treatments was evident, given the almost doubled retranslocation rate of P compared to N. Hence, we conclude that this ecosystem has a high P retention rate, which can contribute to maintain canopy P-stocks over the long term. We suggest that P retranslocation played an important role in this woodland to retain P and enhance its residence time in plants.

### Change in Leaf N and P Concentrations Over Years

Both N and P concentrations varied over the years in this study, for annual values of N_m_ and P_m_ there was some decline in the first 2 years of the experiment (2012–2013), leveling in the last 2–3 years (Figure 1A,B,E,F). This trend was similar or stronger when a consistent leaf age class was selected (i.e., it was not a sampling effect). Some early transients in the N cycle due to elevated CO_2_ have been identified in past studies (Luo et al., 2004), but as indicated in Table 1, the temporal decline we observed in this forest was unrelated to elevated CO_2_, as there was a similar trend in ambient and elevated CO_2_ (Table 1 and Figure 2). Moreover, the elevated leaf N and P concentrations actually preceded the start of the CO_2_ experiment in September 2012. Area-based nutrients (N_a_ and P_a_) also showed the same pattern of cross-year decline, though to a lesser extent (Supplementary Table S1). It is unclear what was responsible for this trend in leaf N and P concentrations given that the trees were mature and the soil at the site remained undisturbed for several decades prior to this study, even during construction of the experimental facility. A possible factor involved in this time trend could have been rainfall, as the elevated CO_2_ experiment was established and begun during a La Niña period, which typically brings rainy conditions to eastern Australia (Chiew et al., 1998). While speculative, higher N and P availability or uptake from the soil may have arisen from more readily available nutrients in the wetter soils during the La Niña period compared to less mobility or reduced uptake during drier periods. This possibility is suggested by the correlations found between leaf P_m_ and N_m_ concentrations and the preceding 6-months rainfall (Supplementary Figure S2). If this speculation is supported by further work, it would indicate that the stand experienced transient higher-nutrient conditions with a rainy period in 2011–2012 but subsequently stabilized leaf N and P concentrations. However, this is still an area for active research.

Regardless of origin, the temporal trend toward a reduction in green leaf N and P concentrations in this mature *Eucalyptus* stand is notable for two reasons. First, it establishes a backdrop to the start of the elevated CO_2_ experiment. Second, it demonstrates that *E. tereticornis* shows flexible N:P rather than maintaining a more conservative stoichiometric homeostasis (Sardans and Peñuelas, 2012). Maintaining stoichiometric homeostasis in environments in the face of variable nutrient supply is energetically demanding for plants (Sterner and Elser, 2002; Dijkstra et al., 2012). However, N:P and C:P ratios in native Australian woodlands seem to have a greater range of values than the C:N ratio, due to smaller variation in N concentrations than P concentrations (see Judd et al., 1996). For instance, *Eucalyptus incrassata* showed a 2-fold variation in N:P across sites with barely any change in C:N (Specht and Rundel, 1990). While the overall response of C:N:P stoichiometry to elevated CO_2_ can significantly vary depending on ecosystem type, climate factors and experimental conditions (Sterner and George, 2000; Castle and Neff, 2009; Yue et al., 2017), the yearly variation in N:P ratios was relatively small in our study. This variation allowed plants to adjust to changing annual conditions while still maintaining their stoichiometry within a fairly narrow range (Elser et al., 2005; Feller et al., 2007; Fitter and Hillebrand, 2009). How flexible leaf and whole-plant stoichiometry are in elevated CO_2_ relative to ambient CO_2_ has important implications for how process-based biogeochemical models represent nutrient limitations that may be manifest in elevated CO_2_ conditions (Zaehle et al., 2014). Thus, the information from this *Eucalyptus* stand establishes a range of flexible foliar N:P values that may be indicative of stoichiometric flexibility in P-limited stands.

### Wood N:P Stoichiometry in Mature Trees

In spite of the lack of CO_2_ treatment-induced changes in leaf stoichiometry over the 5 years, the N:P ratio in sapwood was 36% higher in elevated CO_2_ compared to ambient CO_2_. This was surprising given the advanced age of the mature trees at the site, which were more than 100 years old according to available records. This finding has important implications because wood represents a massive C pool in forest ecosystems, and flexibility in the elemental composition of sapwood can mean greater biomass in elevated CO_2_ without requiring more nutrients. This was not the case in the present study as there was neither a change in biomass accumulation (from Ellsworth et al., 2017) nor a change in sapwood C:P ratio with elevated CO_2_ (Figure 5B).

While this dataset was limited given the destructive nature of the sampling and the need to maintain the long-term integrity of the trees, a higher N:P ratio in wood may indicate an increase in the N supply toward wood relative to P, suggesting storage or accumulation of structural proteins localized in cell walls, or proteins associated with metabolically active parenchyma cells, or both. While not significant, the average wood N concentrations were 20% higher in elevated CO_2_ compared to ambient CO_2_ (Figure 5A) suggesting that the increased N:P ratio of wood in elevated CO_2_ is mainly driven by higher wood N concentrations. Although plants growing in P-limited conditions tend to store a considerable amount of N (Ågren, 2004), the underpinning mechanisms may need to be further investigated. With woody biomass constituting a small P pool (compared to leaves), but a very large carbon pool owing to high C:P ratios (nearly 4000), there may be important implications of stoichiometric flexibility within the sapwood. This magnitude of variable stoichiometry should be considered in large-scale Earth Systems models involving biogeochemistry, as few models currently allow this sort of flexibility (Zaehle et al., 2014).

## Conclusion

This study provided strong evidence of P-limitation in a mature *E. tereticornis* woodland via high foliar N:P ratios and a higher retranslocation of P compared to N on average, supporting our first hypothesis. This is also consistent with our previous findings at the site where P was added to mature trees and enhanced growth relative to unamended trees (Crous et al., 2015). We expected lower leaf N and P concentrations in elevated CO_2_, but for mature leaves there was no significant CO_2_ effect, suggesting these pools were unaltered in the *Eucalyptus* canopy in elevated CO_2_. By contrast, sapwood N:P ratio in elevated CO_2_ increased, suggesting increased N storage in wood, possibly releasing extra CO_2_ as a result of having greater respiratory proteins. Nutrient concentrations and their ratios varied dynamically from year to year depending on varying environmental conditions, with a subtle decline in leaf N and P in both ambient and elevated CO_2_ treatments from the start of the experiment to its fifth year. Thus, the yearly dynamics of nutrient concentration in leaves allowed plants to vary their stoichiometric ratio of N:P to a certain degree. The time-related change in leaf N:P and the CO_2_-treatment induced change in sapwood N:P together suggest that variable nutrient stoichiometry should be accounted for in large-scale Earth Systems models involving biogeochemistry.

## Author Contributions

KC and DE designed the study. KC, DE, AW-K and BM collected the samples with KC performing the P digests and all P analyses as well as N analyses in the first 3 years. AW-K handled the N analyses in the last 3 years. KC wrote the first draft with input from DE, and led the analyses with significant contributions from MJ and DE. All authors commented on this manuscript.

## Conflict of Interest Statement

The authors declare that the research was conducted in the absence of any commercial or financial relationships that could be construed as a potential conflict of interest.

## Footnotes

1 http://www.bom.gov.au
