# Supplementary material for: Nitrogen and Phosphorus Retranslocation of Leaves and Stemwood in a Mature Eucalyptus Forest Exposed to 5 Years of Elevated CO2
Source: Front Plant Sci. 2019 May 31;10:664. doi: 10.3389/fpls.2019.00664 (PMC6554339; doi:10.3389/fpls.2019.00664)
Supplement: Supplementary file 1 [file Data_Sheet_1.docx]

**SUPPLEMENTARY MATERIAL**

The Supplementary Material for the article titled: “**Nitrogen and phosphorus retranslocation of leaves and stemwood in a mature *Eucalyptus* forest exposed to five years of elevated CO_2_” by** Kristine Y. Crous, Agnieszka Wujeska-Klause, Mingkai Jiang, Belinda E. Medlyn and David S. Ellsworth can be found online at: <https://www.frontiersin.org/articles/10.3389/fpls.2019.00664/full#supplementary-material>

**Figure S1| Annual variation in means and standard error plots of leaf chemistry for mature green leaves collected in February (the austral summer) of each year for (A) mass-based phosphorus in mg g^-1^, (B) mass-based nitrogen in mg g^-1^, (C) N:P ratio and (D) C:P ratio (dimensionless).**

**
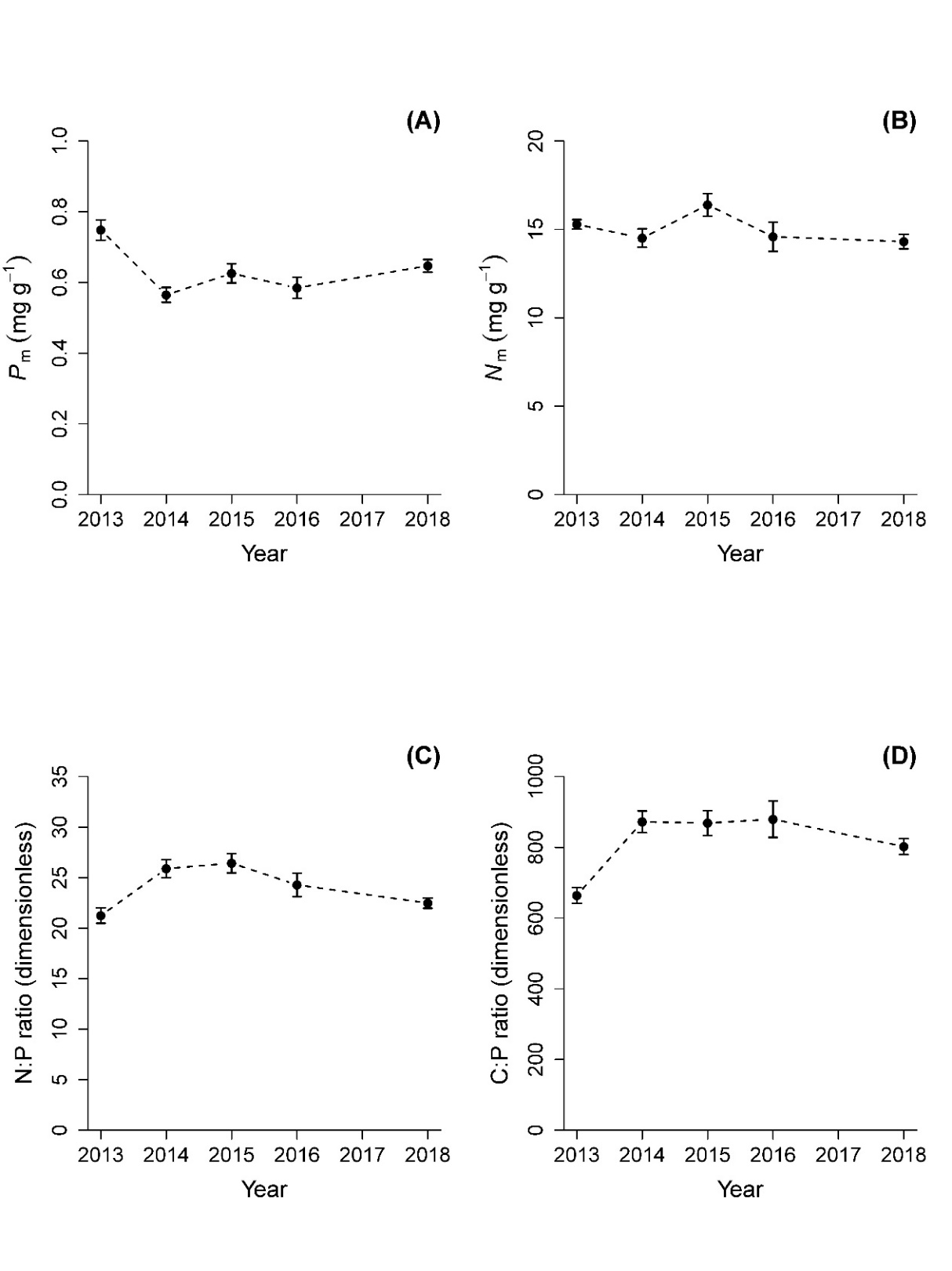
**

**Figure S2: Correlations between mass-based leaf P and N concentration (mg g^-1^) of mature green leaves and the amount of rainfall of the preceding one month (left panels, A and C) and six months (right panels, B and D). Pearson correlation coefficients for each panel respectively were: A) 0.82, B) 0.59, C) 0.56 and D) 0.45. All correlations were significant (*P* < 0.008). Elevated CO_2_ treatment is not indicated as it was not significant (*P* > 0.10).**

**
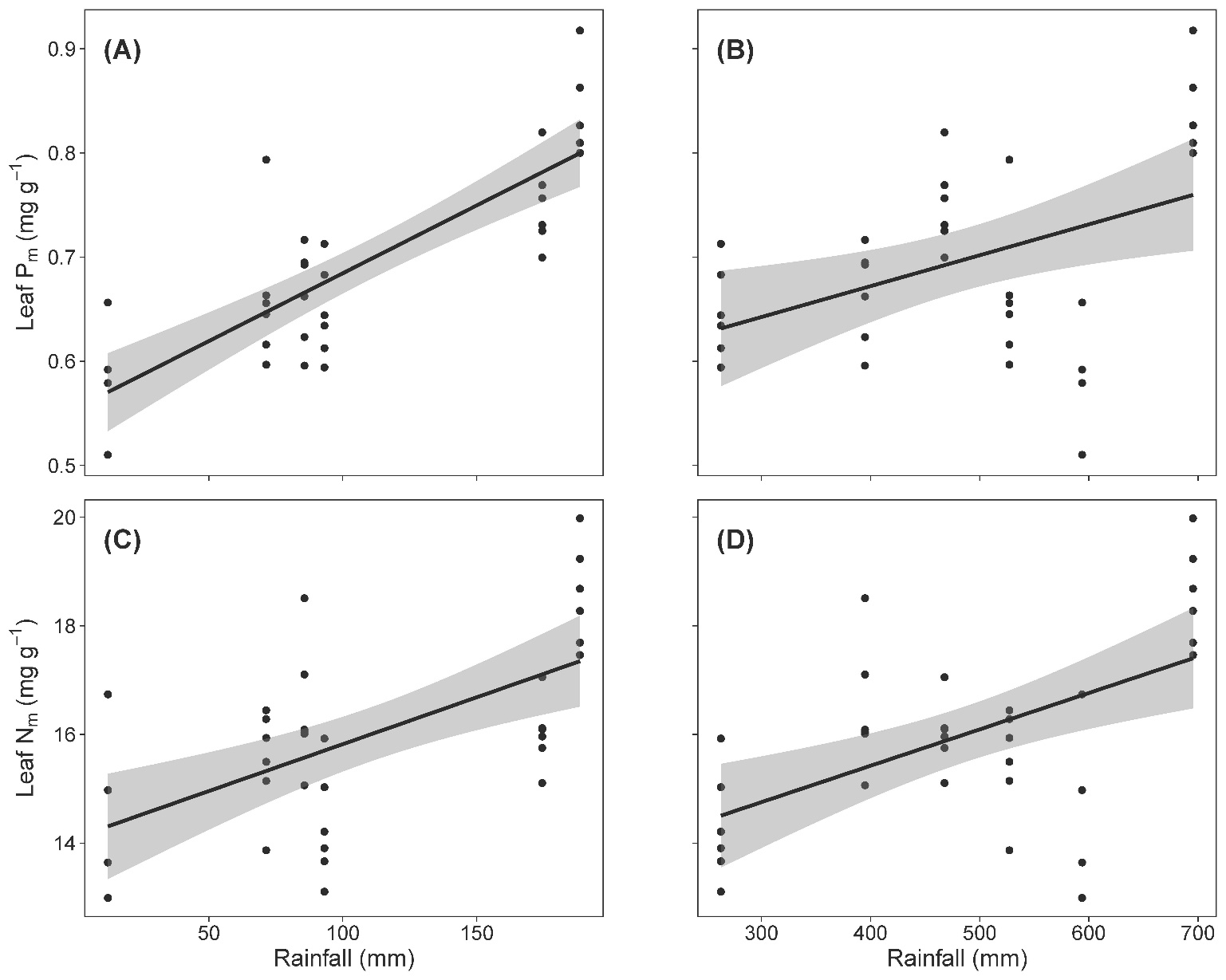
**

**TABLE S1: Mixed model ANOVA table with F-statistic of green upper canopy leaves over five years with CO_2_, Age and Year as fixed factors and tree nested within ring as random factors. df represents the degrees of freedom, where two years did not have the newly-flushed age class resulting in a reduction of df from 5 to 3 for the interactions. The variables are as follows: area-based phosphorus in g m^-2^ (P_a_), area-based nitrogen in g m^-2^ (N_a_) and leaf mass per area ratio in g m^-2^ (LMA). P_a_ was log-transformed for this analysis to conform with normality assumptions. Significant F-statistics are highlighted in bold with significance level indicated as *** for *P* < 0.0001, ** for *P* < 0.01, * for *P* < 0.05 and ^+^ for *P* > 0.05 and *P* < 0.1.**

| **GREEN upper canopy leaves F-statistic and significance** | | | | |
| --- | --- | --- | --- | --- |
| **Source** | **df** | **P_a_** | **N_a_** | **LMA** |
| CO_2_ | 1 | 1.56 | 0.48 | 5.30**^+^** |
| Age | 1 | **20.24***** | **174.2***** | **288.7***** |
| Year | 5 | **20.43***** | **24.24***** | **19.72***** |
| CO_2_ x Age | 1 | 0.07 | 3.35**^+^** | 1.41 |
| CO_2_ x Year | 5 | 2.09**^+^** | 1.19 | 1.78 |
| Age x Year | 3 | **11.88***** | **6.12***** | 0.80 |
| CO_2_ x Age x Year | 3 | 0.81 | 0.19 | 1.20 |
